# Nanomedicines for the Delivery of Antimicrobial Peptides (AMPs)

**DOI:** 10.3390/nano10030560

**Published:** 2020-03-20

**Authors:** Maria C. Teixeira, Claudia Carbone, Maria C. Sousa, Marta Espina, Maria L. Garcia, Elena Sanchez-Lopez, Eliana B. Souto

**Affiliations:** 1 Laboratory of Pharmaceutical Development and Technology, Faculty of Pharmacy, University of Coimbra, Pólo das Ciências da Saúde, Azinhaga de Santa Comba, 3000-548 Coimbra, Portugal; mceuteixeira1@gmail.com (M.C.T.); ccarbone@unict.it (C.C.); 2 Laboratory of Drug Delivery Technology, Department of Drug Sciences, University of Catania, 95131 Catania, Italy; 3 Laboratory of Microbiology, Faculty of Pharmacy, University of Coimbra, Pólo das Ciências da Saúde, Azinhaga de Santa Comba, 3000-548 Coimbra, Portugal; mcsousa@ci.uc.pt; 4 CNC—Center for Neuroscience and Cell Biology, University of Coimbra, 3000-548 Coimbra, Portugal; 5 Department of Pharmacy, Pharmaceutical Technology and Physical Chemistry, Faculty of Pharmacy, University of Barcelona, 08028 Barcelona, Spain; m.espina@ub.edu (M.E.); marisagarcia@ub.edu (M.L.G.); 6 Institute of Nanoscience and Nanotechnology (IN2UB), University of Barcelona, 08028 Barcelona, Spain; 7 Centro de Investigación Biomédica en Red de Enfermedades Neurodegenerativas (CIBERNED), University of Barcelona, 08028 Barcelona, Spain; 8 CEB—Centre of Biological Engineering, University of Minho, Campus de Gualtar, 4710-057 Braga, Portugal

**Keywords:** antimicrobial peptides, bacterial and virus infections, coronavirus, antimicrobial resistance, nanomaterials, nanomedicine

## Abstract

Microbial infections are still among the major public health concerns since several yeasts and fungi, and other pathogenic microorganisms, are responsible for continuous growth of infections and drug resistance against bacteria. Antimicrobial resistance rate is fostering the need to develop new strategies against drug-resistant superbugs. Antimicrobial peptides (AMPs) are small peptide-based molecules of 5–100 amino acids in length, with potent and broad-spectrum antimicrobial properties. They are part of the innate immune system, which can represent a minimal risk of resistance development. These characteristics contribute to the description of these molecules as promising new molecules in the development of new antimicrobial drugs. However, efforts in developing new medicines have not resulted in any decrease of drug resistance yet. Thus, a technological approach on improving existing drugs is gaining special interest. Nanomedicine provides easy access to innovative carriers, which ultimately enable the design and development of targeted delivery systems of the most efficient drugs with increased efficacy and reduced toxicity. Based on performance, successful experiments, and considerable market prospects, nanotechnology will undoubtedly lead a breakthrough in biomedical field also for infectious diseases, as there are several nanotechnological approaches that exhibit important roles in restoring antibiotic activity against resistant bacteria.

## 1. Introduction

The discovery of new antimicrobial molecules in the early 1920s was a landmark in the field of pharmacology allowing the reduction of morbidity and mortality from infectious diseases, which were among the main causes of death worldwide [1]. However, the widespread and indiscriminate use of powerful antibiotics in recent decades has led to a dramatic increase in microbial resistance, being nowadays a major threat to global public health [2]. There is a long list of identified drug-resistant bacteria, including sulfonamide-resistant, penicillin-resistant, methicillin-resistant, macrolide-resistant and vancomycin-resistant, or even multidrug-resistant. As a result, drug-resistant bacterial infections can result in the need to use a higher drug dosage, higher toxicity treatments and longer hospitalization periods, ultimately translated in increased mortality, thus negatively affecting both medicine and society [3]. Antimicrobial resistance is, nowadays, one of the major global economic and healthcare burdens and, despite the efforts in research and development of new molecular entities, the pipeline for new drugs tends to grow on empty [1].

Bacteriocins are peptides produced mainly by Gram-positive bacteria with the main purpose of self-preservation, while a large portion of Gram-negative bacteriocins resemble eukaryotic antimicrobial peptides such as defensins [4]. These antimicrobial peptides depict amphiphilic helices, differing among them in mode of action, biochemical properties and thus spectrum of activity. They are classified according to the host producer, intrinsic function, molecular weight, physicochemical properties and amino acid sequence. Meade et al. published a comprehensive analysis of their classification and use against multi-drug-resistant species [4]. Antimicrobial peptides (AMPs) are small peptide-based molecules, 5–100 amino acids in length, with potent and broad-spectrum antimicrobial properties. AMPs are considered among the most promising drug candidates to be used against infections with the aim to overcome microbial drug resistance. However, they also display some limitations regarding bioavailability and safety, and may possess additional biological activities and functions. AMPs can indeed be used as signaling molecules, in tissue regeneration, as biomarkers and even as tumoricidal agents. AMPs are produced by nearly all living organisms and they act by binding to genetic material, by interacting with the cell wall, cell membrane and/or with intracellular organelles. They act by protecting plants against fungal and bacterial invasions, while in invertebrates and vertebrates they also have activity against parasite and viral invasions which may induce toxic events in the host eukaryotic cells. Table 1 summarizes their main mechanistic pathways. While the mechanisms of action of AMPs have not yet been fully described [5], it is known that AMPs can have receptor-mediated or non-receptor-mediated interactions with the cell membrane [6]. Most of the AMPs produced by bacteria undergo receptor-mediated interactions and are active in vitro at very low concentrations. However, in most vertebrates and invertebrates, AMPs target the membrane without specifically interacting with receptors and are active at concentrations in the micromolar range. They interact with components of the membrane, i.e., with the negative charge of lipids (e.g., lipopolysaccharides, cardiolipin) which attract the positively charged AMPs. Other AMPs may target the bacterial cell wall, inhibit cell wall synthesis or have intracellular targets. While most of the conventional antibiotics compromise the synthesis of cell wall components by binding to specific proteins involved in the reaction, AMPs often interact with precursor molecules needed for the synthesis of the cell wall. As AMPs are produced by several immune cells (e.g., macrophages, neutrophils), the peptides can also activate immune cells which may result in increased microbial killing and/or control of inflammation.

AMPs can also act as delivery systems as an alternative over conventional antibiotics [7], in particular, against nosocomial infections caused by multidrug resistant Gram-negative pathogens [4]. Burrer et al. have used several strains of murine coronavirus in cell culture and in vivo in mouse models for the assessment of the antiviral properties of peptide-conjugated antisense phosphorodiamidate morpholino oligomers (P-PMOs) [8]. The authors have reported the targeting effect of P-PMOs against various target sites in the viral genome in cell culture and protected mice against virus-induced tissue damage. Ykeda et al. have used an antimicrobial peptide isolated from the skin of *Xenopus tropicalis* (Pxt-5), and its modified peptide (Modify-Pxt-5) to produce self-assembled discoidal nanoparticles composed of amphiphilic alpha-helical scaffold proteins or peptides organised in a lipid bilayer [9]. Both the peptides Pxt-5, having hydrophobic and hydrophilic faces, behaved like general surfactants and can be used as carriers. Bacteriocins, while very active at low concentrations, usually have low in vivo stability, being susceptible to degradation by proteolytic enzymes [4]. Another major limitation on the use of these peptides is the difficulty encountered in their large-scale production, compromising altogether their clinical application. An approach to overcome their limited stability in vivo is their loading into nanoparticles.

Nanomedicine is currently a well-established approach intimately related to the design and development of nanomaterials with unique therapeutic and diagnostic properties [10]. Nanotechnologies have also shown great potential in almost every aspect of the management of microbial infection with more than ten nanoparticles (NPs)-based products marketed for bacterial diagnosis, antibiotic delivery and medical devices in 2014. With unique physicochemical characteristics, nanomaterials are sensitive and selective in the detection of bacterial signalling and may also exbibit intrinsic antimicrobial properties. Furthermore, nanomaterials can be used for antimicrobial drug delivery, and the incorporation of antimicrobial nanomaterials in medical devices and implants can prevent microbial adhesion and infection [11,12]. All these facts are instrumental against antimicrobial resistance by compromising bacterial mechanisms of resistance [13]. Focusing on nanomaterials-based drug delivery systems, these offer an improved strategy to increase the therapeutic index, by decreasing the dosage and frequency of administration. Besides, nanomaterials promote intracellular drug delivery, mitigating the development of drug-resistant bacteria and also allowing targeted organ accumulation by functionalized surface modifications, thus limiting systemic side effects and immunosuppression [2]. Despite these promising outcomes, the main challenge of establishing clinical use is related to the evaluation of interactions of nano-antibiotics with cells, tissues and organs achieving information about their possible toxic effects, together with the production on a large scale [1,14,15].

Several types of nanomaterials have shown potential in the pharmaceutical field. They have also been studied as potential drug carriers with applications in the delivery of AMPs, promising antimicrobial molecules which, due to their nature and physicochemical characteristics, have limited bioavailability. The aim of this work is to revise the state-of-the-art on the approach that combines the advantages of the design of new drug delivery systems for the improvement of antimicrobial bioavailability, taking into account the recent developments in nanomaterials for antimicrobial peptide delivery. 

## 2. Antimicrobial Peptides (AMPs): New Additions to the Therapeutic Arsenal

AMPs are small natural oligopeptides that have recently showed a potential activity against antibiotics resistance mechanisms, due to their ability in lysing bacterial membranes, thus providing broad-spectrum effects, targeting microorganisms from viruses to parasites. The main interesting property of AMPs is their ability to selectively disrupt bacterial membranes without affecting mammalian cells, thus being safe. In addition, AMPs are referred to in the literature as host-defence peptides with anti-inflammatory and anticancer activities, because they are synthesized molecules that take action on the defence mechanisms against biological threats of the living organism of origin [16]. The discovery of AMPs dates back to the first half of the 20th century when in 1939, Dubos extracted an antimicrobial agent from a soil *Bacillus* strain, proven to be effective in mice pneumococci infection. This extract was then fractioned allowing the identification of gramicidin. Despite some systemic toxicity, gramicidin has shown to be effective in the topical treatment of wounds and ulcers. The first animal-originated AMP to be reported is defensin, isolated from rabbit leukocytes in 1956 [17]. Nowadays, more than 2000 AMPs have been described and current molecular developments can be consulted in a series of databases available on the web (Table 2), including natural identified molecules, as well as peptidomimetic molecules and analogues that are pharmacologically designed thanks to the use of bioinformatics [18]. Current Food and Drug Administration (FDA)-approved AMPs with well-established use include bacitracin, colistin and polymyxin B (although only for topical administration) [2].

Despite the lack of consensus on the influence of peptide sequence in biologic activity of AMPs, some common characteristics seem to be important and fairly related to their antimicrobial property. The main one is primarily charge, with 90% of AMPs being cationic, and secondly, hydrophobicity or amphipathicity, influencing solubility profiles and consequently bioavailability [19]. Associated with structural characteristics, these are also the main physicochemical properties that should also be taken into account in the design of new synthetic AMPs [17]. Compared to conventional antibiotics, AMPs demonstrate significant advantages such as potency and board spectrum of activity, as well as an additional activity to modulate the immune system responses and low resistance rates. They also show some limitations that impair their safe therapeutic use, such as sensitivity to proteolysis, influencing stability and undefined toxicological data for systemic use [20]. In addition, their cationic and amphiphilic nature lead to high binding to serum proteins after parenteral administration, with consequent rapid elimination from bloodstream circulation and accumulation in the reticuloendothelial system, thus resulting in toxic effects and reduced activity [21]. To overcome these limitations, some strategies have been developed, maximizing the proven therapeutic potential of AMPs. There are numerous methods for obtaining AMP delivery systems. AMPs could be immobilized onto a variety of materials or onto a variety of surfaces and still retain their antibacterial activity. Also, AMPs can be targeted through loading them in nanoparticulate systems with selective delivery capacities, including polymers, liposomes, hydrogels, nanospheres, nano-capsules and carbon nanotubes [19]. As practical examples on of these of strategies, the studies of Mishra et al. were focused on the development of an AMP-coated surface, specifically immobilizing Lassioglossin-III onto silicone-based catheters and its activity against urinary tract infections [22]. At the same time, Water et al. developed the possibility of encapsulating plectasin, a cationic AMP, into polymeric NPs and evaluated their efficacy on *S. aureus* strains [23].

## 3. Nano-Antibiotics: Nanomaterials for Infection Control

Bacteria show resistance to antibiotics drugs through a variety of mechanisms. Moreover, the development of even new mechanisms of resistance has resulted in the simultaneous development of resistance to several antibiotic classes creating very dangerous multidrug-resistant (MDR) bacterial strains [24,25]. However, when bacteria are drug-resistant it does not mean that they stop responding to an antibiotic, but that occurs only at higher concentrations [26,27]. Of greater concern are cases of acquired resistance, where initially susceptible populations of bacteria become resistant to an antibacterial agent, in particular antibiotics, and proliferate and spread under the selective pressure of use of that drug. One approach to address this challenge is to design analogues of drugs [28,29] that are already in clinical use and that have activity against resistant organisms. However, bacteria are constantly succeeding to develop a resistant mechanism to new antibiotic drugs as well as to their analogues [30,31]. 

**Figure 1:**
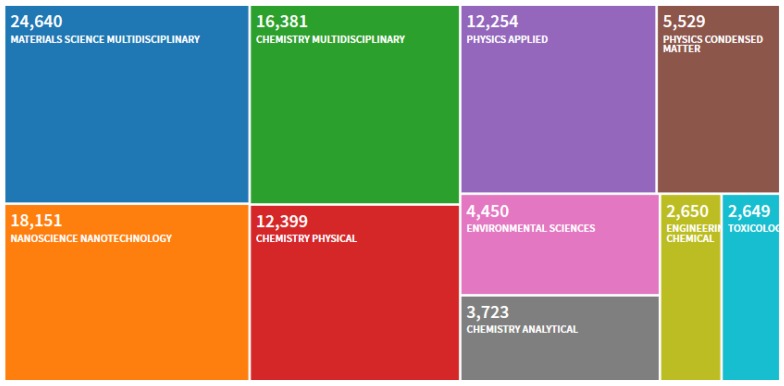
Record count per discipline of the number of papers published between 2000 and 2020 indexed in the Web of Science using “AMPs and Nanoparticles or Nanomaterials” as keywords (date of search; 12th March 2020).

Nanotechnology offers opportunities to re-explore the biological properties of already known antimicrobial compounds such as antibiotics by manipulating their size to modify their effect. The combination of AMPs with nanomaterials is not new. Out of the available 71,102 papers indexed in the Web of Science, a total of 70,948 combining AMPs with nanoparticles or nanomaterials have been published over the last 20 years in a range of disciplines (Figure 1).

**Figure 2:**
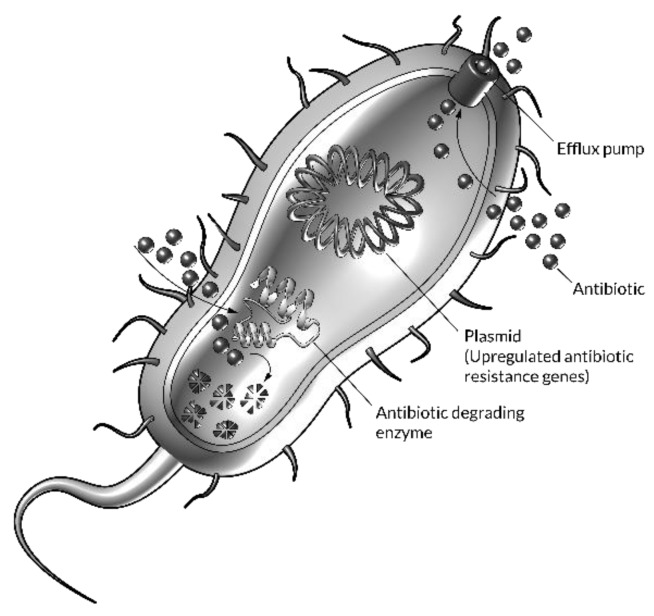
Three of the main antibiotic resistance strategies used by bacteria.

Evidence collected in the review work of Huh and Kwon [1], Pelgrift and Friedman [3], Brooks and Brooks [2], Diab et al. [32] and Shimanovich and Gedanken [33] clarifies microbial drug resistance mechanisms and how nanotechnology may be considered a tool against this issue. Development of drug resistance occurs in (at least) three steps: (i) acquisition by microbes of resistance genes; (ii) expression of those resistance genes; (iii) selection for microbes expressing those resistance genes. First, bacteria acquire resistance to single and multiple drugs through horizontal gene transfer by transformation, conjugation and transduction [34]. Bacteria can also acquire resistance genes by spontaneous mutation of existing genes [35]. MDR is acquired when a bacterial cell already containing one type of drug resistance gene acquires another type of drug resistance gene [34,36]. Second, in response to exposure to an antimicrobial drug, microbes express the resistance gene [36]. Third, resistance becomes widespread when there is selection for microbes that express resistance genes against the antimicrobial drug. This selective pressure in favour of resistance occurs whenever microbes are exposed to the drug but not eradicated (either by the microbicidal effects of the drug itself or by microbiostatic effects of the drug followed by killing by the host’s immune system) [34]. A schematic representation of some specific mechanisms of antimicrobial drug resistance is shown in Figure 2.

**Figure 3:**
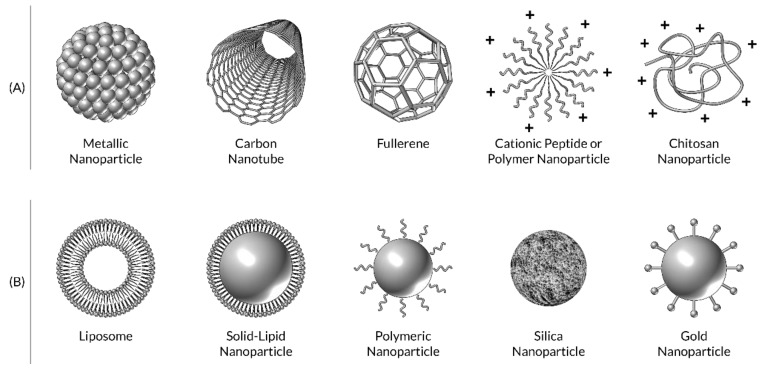
Schematic representation of (**A**) nanomaterials with inherent antimicrobial properties, and (**B**) nanoparticle-based antimicrobial drugs.

Nanomaterials (Figure 3), which either show antimicrobial activity by themselves or elevate the effectiveness and safety of antibiotics administration [10,13], are called “nano-antibiotics” and their capability of controlling infections in vitro and in vivo has been explored and demonstrated. Unlike many antimicrobial agents currently being used in the clinic, antimicrobial NPs may not pose direct and acute adverse effects, although potential toxicity upon long-term exposure is questionable. Most importantly, antimicrobial NPs tackle multiple biological pathways found in broad species of microbes and many concurrent mutations would have to occur to develop resistance against NPs’ antimicrobial activities. Preparation of antimicrobial NPs could be cost-effective, compared to antibiotics synthesis, furthermore they are stable enough for long-term storage with a prolonged shelf-life [37]. In addition, some NPs can withstand harsh conditions, such as high-temperature sterilization, under which conventional antibiotics are inactivated. Antibiotics delivery using nanomaterials offer multiple advantages: (i) controllable and relatively uniform distribution in the target tissue; (ii) improved solubility; (iii) sustained and controlled release; (iv) improved patient-compliance; (v) minimized side effects; and (vi) enhanced cellular internalization [16,37].

## 4. Advances in Nanomaterials for AMP Delivery

Current advances in the use of inorganic nanoparticles for AMP delivery involve essentially the development of silver nanoparticles (AgNPs) and gold nanoparticles (AuNPs), as well as silicon derivates nano-systems [38]. The studies revised in this work are summarized in Table 3. The development of polymeric nanoparticles for AMP delivery may offer an excellent technological strategy to improve drug bioavailability and safety, avoiding drug chemical and enzymatic degradation, preventing aggregation, enhancing controlled release. Chitosan (CS) NPs (CSNPs) are particularly interesting as the broad spectrum of antibacterial activity of CS is well known and documented, offering the possibility of synergistic effects with antimicrobial molecules. Moreover, due to its biocompatibility properties, CS nanostructures have been extensively studied for drug delivery, and that is no different for AMP delivery.

### 4.1. Inorganic or Metallic Nanostructures for AMP Delivery

In recent years, an increasing number of papers reporting on a new generation of antimicrobial metallic NPs has been published [38]. Consequently, many of the information on the application of nanotechnology in the infectious disease field regards the use of silver (Ag) and gold (Au) NPs [11,12,49]. Recently, derivatives of other metals have been studied for antimicrobial applications, and the antibacterial effects of zero-valent bismuth NPs and uncoated Au, nickel (Ni) and silicon (Si) NPs were reported [50,51]. Despite the demonstrated intrinsic antimicrobial properties, dispersed metallic NPs tend to aggregate and separate in solution, resulting in a decrease in their antimicrobial efficiency. With the aim of improving antibacterial properties, functionalization of NPs has been attempted with surfactants, polymers or antibiotics resulting in more stable, less aggregated NPs suspension and innovative, synergistic antibacterial agents. For instance, silver NPs stabilized by polymers(polyvinylpyrrolidone) and surfactants (SDS and Tween 80) exhibit enhanced antibacterial activities [52]. NPs can also act as drug-carriers able to pass through cell membranes [53,54]. Widely used antibiotics such as ciprofloxacin may benefit from the association with NPs, and conjugation may result in an antibacterial effect also against micro-organisms resistant to the same molecule in the naturally occurring form [55]. When the antimicrobial agents are covalently linked to or contained within, NPs, a higher drug concentration is attained in the area of interest, resulting in better efficacy at comparable doses and in slower release over time that may be exploited for preventing bacterial colonization [56,57]. Moreover, specific biological sites can be attacked after modification of NPs with target molecules [58,59]. As the NPs themselves may have antibacterial properties, the combination of NPs and loaded drugs exert a synergistic action [60]. Current advances in the use of inorganic nanostructures for AMP delivery involve essentially the development of Ag and AuNPs, as well as silicon derivate nano-systems. The studies revised in this work are summarized in Table 3.

#### 4.1.1. Gold Nanoparticles

Research on gold NPs is increasing thanks to their many advantages, such as their ease of synthesis and conjugation to biomolecules, their capability to maintain their own structure when in circulation and their improved effectiveness against bacteria, thus demonstrating their high potential in the field of nanomedicine. Even if at the beginning, the research in this field was focused on the possibility to exploit gold NPs in combination to laser radiation, thus significantly reducing bacteria viability due to cell lysis and mechanical disruption [61]. Currently, two recent studies have been reported exploring the use of Au nanostructures for AMP delivery. Photoluminescent Au nanodots (AuNDs) were prepared by Chen et al. [39]. These AuNDs were functionalized with hybridized ligands, an antimicrobial peptide (surfactin; SFT), and 1-dodecanethiol (DT), on AuNPs. Ultrasmall SFT/DT–Au NDs (size ≈ 2.5 nm) were achieved and exhibited highly efficient antimicrobial activity. The photoluminescence properties and stability as well as the antimicrobial activity of SFT/DT–Au NDs were also studied, and it was shown that these characteristics are highly dependent on the density of SFT on Au NDs. Relative to SFT, SFT/DT–AuNDs exhibited greater antimicrobial activity, not only to non-multidrug-resistant bacteria but also to the multidrug-resistant bacteria. The minimal inhibitory concentration values of SFT/DT–AuNDs were much lower (>80-fold) than that of SFT. The authors considered that the antimicrobial activity of SFT/DT–AuNDs was mainly achieved by the synergistic effect of SFT and DT–AuNDs on the disruption of the bacterial membrane. In vitro cytotoxicity and hemolysis, analyses were also performed and had revealed superior biocompatibility of SFT/DT–AuNDs than that of SFT. Moreover, in vivo methicillin-resistant *S. aureus* infected wound healing studies in rats showed faster healing, better epithelialization. This study suggested that the SFT/DT–AuNDs system may be a promising antimicrobial candidate for preclinical applications in treating wounds and skin infections [39]. Rai et al. also reported a one-step methodology to generate AMP-conjugated (AuNPs) [40]. The AMP-conjugated AuNPs prepared showed controlled size (14 nm) and low polydispersity and allowed the inclusion of high concentration of AMPs. Further, these systems demonstrated higher antimicrobial activity and stability in serum and in the presence of non-physiological concentrations of proteolytic enzymes than soluble AMP, as well as low cytotoxicity against human cells [40]. Interestingly, Akrami et al. showed the possibility to exploit gold NPs as a novel anticancer platform [41]. Results of this research confirm the improvement of cell internalization of gold NPs, with higher cytotoxicity and cellular uptake for smaller NPs compared to larger nanospheres and nanorods, suggesting that anticancer effects of the selected peptides were modulated by the size and shape of the gold nanoparticles [41].

#### 4.1.2. Silver Nanoparticles

Geilich et al. developed novel delivery systems by combining silver NPs and ampicillin so to achieve a synergistic dose-dependent effect on bacterial cells [42]. The obtained polymersomes were safe on human fibroblasts and more effective in inhibiting bacterial cells with a silver-to-ampicillin ratio of one to 0.64, respectively. Recent advances in AMP delivery by Ag nanostructures, Pazos et al. [43] reported on supramolecular assemblies of novel peptide amphiphiles (PAs) containing aldehyde functionality in order to reduce Ag ions and subsequently nucleate Ag metal NPs in water. This proposed system spontaneously generates monodisperse Ag particles at regular distances along the length of the filamentous organic assemblies. The metal−organic hybrid structures exhibited antimicrobial activity and significantly less toxicity toward eukaryotic cells. Metallized organic nanofibers of the type described offer the possibility to create other structures. For instance, hydrogels, that can be potentially applied in wound dressing development [43]. Also addressing the wound infection problem, Salouti et al. investigated the synergistic antibacterial effect of plant peptide MBP-1 and AgNPs on infected wounds caused by *S. aureus* [44]. The MIC and MBC of MBP-1 and AgNPs both on their own and in combination form were determined against *S. aureus* via macrodilution and microdilution methods. The MIC and MBC of MBP-1 were found to be 0.6 and 0.7 mg/mL, respectively. MIC and MBC of AgNPs were determined to be 6.25 and 12.5 mg/L, respectively. MIC and MBC of the AgNPs and MBP-1 combination were found to be 3.125 mg/mL, 0.5 mg/L; and 6.25 mg/mL, 0.6 mg/L, respectively. The synergistic antibacterial effect of Ag NPs and MBP-1 was investigated on infected wounds caused by *S. aureus* in a mouse model, and the infected wound healed properly after the combined use of MBP-1 and AgNPs [44]. It is worth to note recent findings by Chaudhari et al. concerning the conjugation of silver-coated carbon nanotubes with the antimicrobial peptide TP359. Herein, authors underline the occurrence of an additive effect of silver-coated nanotubes and TP359 regarding antibacterial activity. The innovative nano-system was found to be safe to murine macrophages and Hep2 cells at the MIC concentrations [45].

#### 4.1.3. Silicon Nanostructures

As delivery systems for AMPs, silicon and silicon derivates nanostructures have also been investigated recently. Membrane interactions are critical for the successful use of mesoporous SiNPs. In order to elucidate these, Braun et al. have studied the effects of NP charge and porosity on AMP loading and release, as well as consequences of this for membrane interactions and antimicrobial effects [46]. Anionic mesoporous SiNPs were found to incorporate considerable amounts of the CAMP LL-37, whereas loading was found to be much lower for non-porous or positively charged SiNPs. The results also demonstrated that due to preferential pore localization, anionic mesoporous particles, but not the other particles, protects LL-37 from degradation by infection-related proteases. For anionic SiNPs, membrane disruption is mediated almost exclusively by peptide release. In contrast, non-porous SiNPs built up a resilient LL-37 surface coating due to their higher negative surface charge, and display largely particle-mediated membrane interactions and antimicrobial effects. For positively charged mesoporous SiNPs, LL-37 incorporation promoted the membrane binding and disruption displayed by the particles in the absence of peptide, but also caused toxicity against human erythrocytes. Thus, the use of mesoporous SiNPs as AMP delivery systems requires consideration of membrane interactions and selectivity of both free peptide and the peptide-loaded NPs [46]. The properties of AMPs adsorbed on inorganic or organic surfaces are of interest for their potential applications in intracellular drug delivery. In the work of Syryamina et al., continuous-wave (CW) electron paramagnetic resonance (EPR) and pulsed electron-electron double resonance (PELDOR) techniques were applied to study the adsorption of the AMPs trichogin GA IV and ampullosporin A on monodisperse colloidal silica nanospheres (SiNS) of 20 nm diameter [47]. The results obtained by CW EPR supported the view that the adsorbed peptides form close-packed clusters. PELDOR data show that both trichogin and ampullosporin adsorbed on the silica surface possess a more disordered conformation as compared to that in solution. For ampullosporin, disordering is much more pronounced than for trichogin. After desorption, the peptides restored their conformations; upon adsorption, the peptides in some cases may lose partly their biradical character [47]. These results may be of interest as the antimicrobial activity is often related to peptide conformation.

Nano-clays or layered silicates are an interesting nanostructure that has been used for remediation of environmental contaminants, delivery of drugs and various active molecules, and to enhance polymer mechanical and barrier properties in packaging films. They typically present a stacked arrangement of silicate layers with a nanometric thickness [62]. Meira et al. studied three different nano-clays (bentonite, octadecylamine-modified montmorillonite, and halloysite) as potential carriers for the AMPs nisin and pediocin, known bacteriocins, the first referred above as having application as a food preservative. Higher adsorption at room temperature of nisin and pediocin was obtained on bentonite. The antimicrobial activity of the resultant bacteriocin-nano-clay systems was analysed using skimmed milk agar as food simulant, and the largest inhibition zones were observed against Gram-positive bacteria for halloysite samples. Bacteriocins were intercalated into the interlayer space of montmorillonites as deduced from the increase of the basal spacing measured by X-ray diffraction (XRD) assay. These results indicate that nano-clays, especially halloysite, are suitable nano-carriers for nisin and pediocin adsorption, and the results may be considered interesting for the food industry [48].

### 4.2. Polymeric Nanoparticles for AMP Delivery

Different biodegradable polymeric nano-systems have been explored as carriers for antimicrobial agents that exhibit a high bactericidal activity. The efficacy of this strategy is well proven, highlighting polymeric nano-systems can effectively improve the cellular penetration, intracellular retention and specific subcellular distribution of antimicrobial agents, and even evade intracellular inactivation of antimicrobial agents [63]. 

Controlled drug release using biocompatible and biodegradable polymers further emerged in the 1980s [64]. After the first polymer-based delivery of macromolecules using poly[ethylene vinyl acetate] polymer was demonstrated in 1976 [65,66]. Antimicrobial drug delivery using polymeric NPs offers several advantages: (i) structural stability in biological fluids and under harsh and various conditions for preparation; (ii) precisely tuneable properties, such as size, zeta-potentials, and drug release profiles, by manipulating polymer lengths, surfactants, and organic solvents used for NP preparation [67], and (iii) facile and versatile surface functionalization for conjugating drugs and targeting ligands [68]. 

Concerning antimicrobial nanoparticulate delivery systems, two major types of polymers have been explored: linear polymers (e.g., polyalkyl acrylates and polymethyl methacrylate) and amphiphilic block copolymers. The majority of polymeric NPs prepared with linear polymers are nano-capsules or solid nanospheres [69]. In polymeric nano-capsules, a polymeric membrane that controls the release rate surrounds the drugs that are solubilized in aqueous or oily solvents. In solid nanospheres, drugs are homogeneously distributed in the polymeric matrices of variable porosities [70,71]. Amphiphilic block copolymers form self-assemble micellar NPs with the drug being encapsulated in the hydrophobic core and surrounded by a hydrophilic shield. This shied allows the core to be protected from degradation [72]. Several biodegradable polymers, including poly(lactic acid) (PLA), poly(glycolic acid) (PGA), poly(lactide-co-glycolide) (PLGA), poly(caprolactone) (PCL), and poly(cyanoacrylate) (PCA), have been used as the hydrophobic core of the amphiphilic copolymers, whereas PEG has been the most commonly used hydrophilic segment [67,73,74,75,76]. Targeting ligands can also be conjugated on the termini of PEG for targeted and selective delivery [77,78]. 

Polymeric NPs have been explored to deliver various antimicrobial agents and greatly enhanced therapeutic efficacy in treating many types of infectious diseases has been reported. For instance, encapsulated ampicillin in polymeric NPs was effective for *S. typhimurium* infection treatment [79] and intracellular L. monocytogenes infection in mouse peritoneal macrophages [80]. The use of polymeric NPs can overcome the limited oral administration of unstable or inadequately absorbed drugs, [81] and, in addition, PEGylation of NPs, can increase drug half-life in serum, and improve mucoadhesive capabilities by reducing phagocytosis [82] Thus, among nanoparticle platforms, polymer NPs may be the most suitable system that can be used for antimicrobial drug delivery. 

Biodegradable polymers and bioorganic polymers are also promising materials in the delivery of peptide-based drugs due to their compatibility, degradation behaviour, and nontoxic nature of administration [83]. The development of polymeric therapeutic nanostructures for AMP delivery may offer an excellent technological strategy to improve drug bioavailability and safety. CS-based NPs (CSNPs) are particularly interesting as the broad spectrum of antibacterial activity of CS is well known and documented, offering the possibility of synergistic effects with antimicrobial molecules. Moreover, due to its biocompatibility properties, CS nanostructures have been extensively studied for drug delivery, and that is no different for AMP delivery. In fact, the majority of the studies performed so far involves CS nanostructures and was already revised in previous work [84] from which we reproduce Table 4. Research has been covering, the technological development of new carrier systems and their full characterization, and the evaluation of their efficacy as drug delivery improvers. In addition to CS-based nanostructures, recent studies on other polymeric nanostructures for AMP delivery have been developed (Table 5).

D’Angelo et al. designed and developed a system of nano-embedded microparticles (NEM) for sustained delivery of cationic AMPs (CAMPs) [94] in the lung, studying its effect on P. aeruginosa, a known lung infection pathogen. To this purpose, PLGA NPs containing a model CAMP, colistin (Col), were produced by the emulsion/solvent diffusion technique, and then spray-dried in different carriers (lactose or mannitol), thus producing NEM. The most promising NEM formulations were selected from bulk and flow properties, distribution of NPs in the carrier and aerosolization performance upon delivery through a breath-actuated dry powder inhaler. Col–loaded NEM were found to kill P. aeruginosa biofilms and to display a prolonged efficacy compared to the free Col. [94]. Another CAMP, plectasin, was encapsulated into PLGA NPs using the double emulsion solvent evaporation method, in the work of Water et al. [23] The plectasin-loaded NPs displayed a high encapsulation efficiency (71–90%) and mediated release of the peptide over 24 h. The antimicrobial efficacy was investigated using bronchial epithelial Calu-3 cell monolayers infected with *S. aureus*, and encapsulated plectasin displayed improved efficacy as compared to non-encapsulated plectasin. The author also assessed the subcellular localization of the prepared NPs in different relevant cell lines: Calu-3 epithelial cells, THP-1 macrophages and A549 epithelial cells. Here the results have shown good patterns of penetration on Calu-3 epithelial cell lines, as well as in THP-1 macrophages [23].

Hydrogels and nanogels are an important class of biomaterials that have been widely utilized for a variety of biomedical/medical applications. The biological performance of these systems, particularly those used as wound dressing; can be complemented with antimicrobial activity capable of preventing colonization of the wound site by opportunistic bacterial pathogens [90,100,101]. These types of structures have also been studied recently for AMP delivery. Continuing their study of the antimicrobial activity of multi-domain CAMPs (MD-CAMPs) in solution, Jiang et al. investigated the same effect of self-assembled 3-D hydrogels supramolecular nanostructures and its rheological properties [95]. Among the studied MD-CAMPs solutions, the bactericidal activity of peptide hydrogels was found to be improved. The improved antimicrobial activity of the self-assembled peptide hydrogels was found to be related to the combined effect of supramolecular surface chemistry and storage modulus of the bulk materials, rather than the ability of individual peptides/peptide assemblies to penetrate bacterial cell membrane as observed in solution. Thus, the structure–property–activity relationship developed through this study may provide important knowledge for designing biocompatible peptide hydrogels with built-in antimicrobial activity for various biomedical applications [95]. The Water et al. group also designed novel nanogel-based novicidin delivery system. The peptide novicidin was self-assembled with a nano-ctenyl succinic anhydride-modified analogue of hyaluronic acid, and this formulation was optimized using a microfluidics-based quality-by-design approach. The encapsulation efficiency of novicidin (15–71%) and the zeta potential (−24 to −57 mV) of the nanogels could be tailored by changing the preparation process parameters, with a maximum peptide loading of 36 ± 4%. The nanogels exhibited good colloidal stability under different ionic strength conditions and allowed complete release of the peptide over 14 days. Furthermore, self-assembly of novicidin with hyaluronic acid into nanogels significantly improved the safety profile at least five-fold and six-fold when tested in HUVECs and NIH 3T3 cells, respectively, while showing no loss of antimicrobial activity against *E. coli* and *S. aureus* [96]. Li et al. explored the potential application of AMPs in wound healing, by developing a biodegradable poly(L-lactic acid)-Pluronic L35-poly(L-lactic acid) (PLLA-L35-PLLA) in situ gel-forming system [97]. An injectable formulation composed of human AMPs 57 (AP-57) loaded NPs, and thermosensitive hydrogel was prepared. AP-57 peptides were enclosed with biocompatible NPs (AP-57-NPs) with high drug loading and encapsulation efficiency. AP-57-NPs were further encapsulated in a thermosensitive hydrogel (AP-57-NPs-H) to facilitate its application in cutaneous wound repair. As a result, AP-57-NPs-H released AP-57 in an extended period and exhibited quite low cytotoxicity and high anti-oxidant activity in vitro. The in vivo wound healing assay using a full-thickness dermal defect model of SD rats indicated that AP-57-NPs-H could significantly promote wound healing. At day 14 after an operation, the treated group showed nearly complete wound closure of 96, 78 ± 3, 12% [97]. Other studies of nisin nanoencapsulation were performed, with the purpose of protection to ensure the stability of this AMP during food processing and storage period. Nisin-loaded pectin NPs (NLP-NPs) were prepared and analysed by Krivirotova et al. by a simple complexation method [98]. Three types of pectin biopolymer were tested and it was found that the methoxylation degree of pectin influenced nisin loading efficiency and particle size. For the complex formation, both electrostatic and hydrophobic interactions were important. NLP-NPs exhibited antimicrobial activity that was dependent on the type of biopolymer. Overall, the results indicated that NLP-NPs might be a suitable antimicrobial system to be used in the food industry [98]. Nisin nanoencapsulation in self-assembly chitosan- and poly- glutamic acid, was recently reported by Wu et al. [99]. Herein, the authors showed that the use of a coating layer of chitosan improved colloidal stability, loading capacity and encapsulation efficiency. Furthermore, chitosan layer composite nanoparticles were more effective compared to uncoated NPs and nisin in inhibiting the growth of *Escherichia coli* and *Listeria monocytogenes.*

### 4.3. Lipid Nanoparticles for AMP Delivery

Solid lipid nanoparticles (SLN) and nanostructured lipid carriers (NLC) stand for nanoparticles composed of lipid materials that melt at temperatures above 40 °C [102]. Their main purpose is to modify the release profile of the payload attributed to their solid core. While SLN are composed of solid lipids only, NLC matrix is a blend of solid and liquid lipids (which also melts above the body temperature) to improve the solubility of lipophilic compounds. Both SLN and NLC are biodegradable and non-toxic [103,104,105], and their versatility of is also attributed to their capacity to load a range of chemically different bioactives, including peptides and proteins [106,107,108,109], and be modified on their surface [110,111]. Fumakia et al. have developed SLN for the simultaneous delivery of an endogenous host defence peptide (LL37) with antimicrobial activity and an elastase inhibitor serpin A1 (A1) for the treatment of wound infections [112]. The authors reported that SLN could modify the release profile of simultaneous delivery of both bioactives, accelerate wound healing in BJ fibroblast cells and keratinocytes and promote antibacterial activity against *Staphylococcus aureus* and *Escherichia coli* in comparison to LL37 or A1 alone. 

LL37 loaded into NLC have also been proposed by Garcia-Orue for the topical treatment of chronic wounds [113]. LL37-loaded NLC did not affect cell viability tested against human foreskin fibroblasts while the in vitro bioactivity assay showed that the peptide remained active after the loading into lipid nanoparticles as the system reversed the macrophages activation as happened with the LL37 in solution. LL37-loaded NLC were also active against Escherichia coli. The in vivo testing in mice also demonstrated the systems’ capacity to improve healing by promoting wound closure, reepithelization grade and restoration of the inflammatory process.

Lewies et al. demonstrated that biodegradable NLCs enhance the antibacterial activity of antimicrobial peptides [114]. The authors have studied the effect of nisin Z when loaded into nanostructured lipid carriers on *Staphylococcus aureus, Staphylococcus epidermidis* and *Escherichia coli*. Nisin Z exhibited additive interactions with numerous conventional antibiotics, in particular, with novobiocin. The presence of EDTA improved the antimicrobial activity of free nisin Z towards *Escherichia coli* significantly. Nisin Z-loaded NLCs were effective against Gram-positive species at physiological pH, also showing an increased efficacy when adding EDTA. Nisin Z-loaded NLCs were shown potential to enhance the antibacterial activity of nisin Z towards Gram-positive bacteria commonly found in skin infections.

### 4.4. Other Nanostructures for AMP Delivery

Other types of nanomaterials, such as dendrimers and carbon nanodots, have also been successfully proposed for the delivery of AMPs. Due to their ease of synthesis and low manufacturing costs, antimicrobial polymers including dendrimers have been exploited to mimic the antibacterial mechanism host defence peptides, by compromising bacterial cell membranes [115]. Gide et al. developed nanomaterials-lipidated amphiphilic dendrimers which displayed potent and selective antimicrobial activity against both Gram-positive and Gram-negative bacteria, including multidrug-resistant strains [115]. These dendrimers are also shown to inhibit bacterial biofilms effectively.

Carbon nanodots or carbon quantum dots were originally discovered due to their bright fluorescence emissions as those found in conventional semiconductor quantum dots, and can also be used for drug delivery [116]. Carbon dots modified with vancomycin were proposed for the treatment of Gram-positive bacterial infections [117]. Zhong et al. synthesized surface-modified carbon dots with vancomycin and successfully tested them against *Bacillus subtilis, Listeria monocytogenes, Salmonella, Pseudomonas aeruginosa* and *Escherichia coli*. The modified carbon dots showed affinity to the tested Gram-positive bacteria owing to the ligand–receptor interactions between vancomycin and the cell walls. Pramanik et al. synthesis red/blue fluorescent carbon dot-attached magnetic nanoparticles for selective separation and identification of superbugs from infected blood samples [118]. The nanoparticles were capable of isolating methicillin-resistant *Staphylococcus aureus* (MRSA) and *Salmonella* DT104 superbug from whole blood samples, followed by accurate identification via multicolor fluorescence imaging. The authors also described the design of antimicrobial peptide-conjugated multicolor fluorescent magneto-carbon dots for separation, identification and disinfection of multidrug resistance superbugs from infected blood.

## 5. Conclusions and Future Challenges

AMPs are antimicrobial compounds recognized as among the most promising drug candidates against infections. They exhibit distinct mechanisms of action and may show additional biological activities, e.g., as signalling molecules and biomarkers, and even as tumoricidal agents. AMPs, however, show low stability and bioavailability. The formulation of AMPs into nanomaterials seems to offer a very appealing and effective manner to improve these limitations, and several systems have been designed and studied for this purpose (e.g., inorganic, polymeric and lipid nanoparticles, carbon dots and dendrimers). However, concerns on how to regulate the distribution of nanomaterials in the body or specific organs are also raised. Nano-drugs are foreign substances to the body and may produce inflammation. Therefore, safety data for long-term therapy or repeated dosage are needed to circumvent the potential risk. Biodegradable nanomaterials have been proposed to reduce the risk of toxicological events both in vitro and in vivo. To date, few studies have investigated the toxicological and environmental effects of direct and indirect exposure to nanomaterials, and no clear guidelines exist to quantify these effects. Therefore, there is an urgent need for developing guidelines, which can assure the safe use of nanomaterials. Moreover, more powerful ex vivo models or animal models are needed to assess the safety issues and to comply with government regulations. How to extend the shelf life of nanodrugs is also a problem due to their agglomeration also being a problem. The production methods for nanostructures should also be improved, and scalable studies for industrial production are also of great importance in order to promote cost effectiveness of these new formulations. The cost and production of nanomaterials on a large scale is one of the hurdles in effective implementation of these products. Hence, the scientific community should also pay attention to developing affordable methodologies so that nanotechnology can reach patients. In conclusion, it seems that, although the promising research results in this area are rising, it is also urgent to start directing efforts in making these new drug formulations a reality as therapeutic agents.

## Figures and Tables

**Table 1:** Mechanisms of action of antimicrobial peptides.

AMPs Action	Effects	
Membrane-targeting (direct cell killing)	Cell membrane disruption	
	Specific binding to membrane receptors	
Nonmembrane-targeting (immune modulation)	Activation of immune cells	Controlled inflammation
		Increased killing and clearance of pathogenic microorganisms

**Table 2:** Currently available AMPs databases.

Database	Description
Collection of antimicrobial peptides (CAMP)	Holds experimentally validated and predicted AMP sequences
AMPer	Database and automated discovery tool for gene-coded AMPs
Antimicrobial Peptide Database (APD)	Contains mostly AMPs from natural sources (~98% of the entries)
Yet Another Database of Antimicrobial Peptides (YADAMP)	Mostly focused on bacterial AMPs
BACTIBASE	Data repository of bacteriocin AMPs
PhytAMP	Database dedicated to antimicrobial plant peptides
RAPD	Database containing recombinantly-produced AMPs
HIPdb	Experimentally validated HIV inhibitory peptides
Bagel2	Bacteriocin mining tool
Peptaibol	Database for peptaibols (unusual peptides)
PenBase	Database devoted to penaeidins
Defensins Knowledge Base	Information and database dedicated to defensins
CyBase	Database specialized in cyclotides

**Table 3:** Recent studies on inorganic nanoparticles for AMP delivery.

System	AMP	Study Description	Authors
**Au nanoparticles**			
*Photoluminescent gold nanodots (AuNDs)*	Surfactin	AuNDs functionalized with hybridized ligands, an antimicrobial peptide (surfactin; SFT), and 1-dodecanethiol (DT), on Au NPs, for application in wound infection treatment.	Chen et al. [39]
*AuNPs*	Cecropin melittin Magainin-1 Tet-20	Development of a one-step synthetic route form functionalization of AuNPs with AMPs	Rai et al. [40]
*AuNPs*	Anti-apoptotic peptide	Modulation of the anti-cancer effects of the selected peptides	Akrami et al. [41]
**Ag nanoparticles**			
*AgNPs*	Ampicillin	Polymersomes for the conjugation of AgNPs with ampicillin, to achieve synergistic effects	Geilich et al. [42]
*AgNPs nanofiber*	PAs	Study of metallized organic nanofibers for application in wound infection treatment	Pazos et al. [43]
*AgNPs*	MBP-1	Study of the synergistic antibacterial effect of plant peptide MBP-1 and AgNPs on infected wounds caused by *S. aureus*	Salouti et al. [44]
*Ag-coated nanotubes*	T 359	Study of additive antimicrobial effects between nanotubes and AMP T 359	Chaudhari et al. [45]
**Si nanoparticles**			
*Anionic mesoporous SiNPs*	LL-37	Study of membrane interaction with AMPs in different types of mesoporous particles.	Braun et al. [46]
*Nanospheres*	Trichogin GA IV Ampullosporin A	Study of AMPs properties adsorbed on silica-based surfaces for potential applications in intracellular drug delivery	Syryamina et al. [47]
*Nano-clays*	NisinPediocin	Study of nano-clays as nano-carriers for nisin and pediocin adsorption, for applications in food industry.	Meira et al. [48]

**Table 4:** Recent studies on CS based nanoparticles for AMP delivery.

System	AMP	Study Description	Authors
**NPs**			
*CS-alginate nano-capsules*	-	Development of nano-capsules carriers for bioactive compounds, produced through LbL technique using, 5-aminosalycilic acid and glycomacropeptide model.	Rivera et al. [85]
*CS-based nanoparticles*	Vancomycin	CS particles were prepared by ionic gelation and freeze-drying or spray-drying as recovery methods. Antibacterial activity against *S. aureus.*	Cerchiara et al. [86]
*CS-based nanoparticles*	Lysozyme as model	Development of a nanoparticle model with commercially available CS loaded with lysozyme as antimicrobial protein drug model.	Piras et al. [87]
*CS and poly-gamma-glutamic acid composites*	LL-37	The results indicated that both LL-37 and NO were co-loaded successfully in micro particles, and the composite particles could sustain LL-37 and NO release at physiological pH, *in vitro.*	Sun et al. [88]
*CS tripolyphosphate (CS-TPP) nanoparticle*	Cryptdin-2	Preparation of CS tripolyphosphate (CS-TPP) NPs by ionotropic gelation. The formulation was then characterized on the basis of particle size, zeta potential and polydispersity, and antimicrobial in vivo assays against *Salmonella enterica* were performed.	Rishi et al. [79]
*CS-based nanoparticles*	Temporin B	CS-NPs were prepared based on the ionotropic gelation between CS and sodium tripolyphosphate. The nano-carrier evidenced a sustained antibacterial action against various strains of *S. epidermidis.*	Piras et al. [89]
**Nanogels**			
*Nanogel composites*	-	Prepation of AgNPs embedded in a biocompatible nanogel comprising degradable, natural polymers. In this study, hybrid nanogels were prepared with varying polymer content and their potential by determining their antibacterial properties against *E. coliand S. aureus* strains.	Coll Ferrer et al. [90]
*Glycol- CS nanogels*	-	Study of the biocompatibility of a glycol CS nanogel by evaluation of effects on metabolic activity, cell cycles blood compatibility. Overall, the results demonstrated the safety of the use of the GC nanogel as drug delivery system.	Pereira et al. [91]
**Nanofibers and films**			
*CS thin films*	hLF1-11	Immobilization performed onto CS thin films as a model for an implant coating due to its reported osteogenic and antibacterial properties. CS thin films were produced by spin-coating on Au surfaces. Activity against methicillin-resistant *S. aureus* (MRSA).	Costa et al. [92]
*Nanofibers*	Defensin-1, Dermaseptin LL-37 Magainin 1	Alternate deposition of polycation (CS) and polyanion over cotton gauzes. Antimicrobial assays were performed with two strains: *S. aureus* and *K. pneumonia*.	Gomes et al. [92]
**Food packaging systems**			
*CS films*	Nisin	Study of the efficiency as antimicrobial carriers of hydroxypropyl methylcellulose [93], chitosan (CS), sodium caseinate (SC) and polylactic acid [93] films, in the release rates of fluorescently labeled nisin Z, evaluating their potential as food packaging polymers.	Imran et al. [93]

**Table 5:** Recent studies on other polymeric nanoparticles for AMP delivery.

System	AMP	Study Description	Authors
**NPs**			
*PLGA nanoparticles*	Colistin	Development of a system of nano-embedded microparticles (NEM) for sustained delivery of CAMPs in the lung	D’Angelo et al. [94]
*PLGA nanoparticles*	Plectasin	Intracellullar antibacterial activity against *S. aureus* in epithelial cells.	Water et al. [23]
**Hydro- and Nanogels**			
*Self-assembled 3-D hydrogels*	MD-CAMPs	Study of bactericidal activity compared to MD-CAMPs in solution, and rheological properties.	Jiang et al. [95]
*Hyaluronic acid nanogel*	Novicdin	Quality-by-design novel nanogel-based novicidin delivery system.	Water et al. [96]
*PLLA-L35-PLLAin situ gel*	AP-57	Potential application of AMPs in wound healing, by developing a biodegradable poly (L-lactic acid)-Pluronic L35-poly (L-lactic acid) (PLLA-L35-PLLA) in situ gel-forming system	Li et al. [97]
**Food preservation systems**			
*Pectin nanoparticles*	Nisin	Study of a safe suitable antimicrobial system to be used in food industry. Influence of pectin degree of acetylation on NP properties.	Krivorotova et al. [98]
*Chitosan/PGA nanoparticles*	Nisin	Influence of chitosan coating on colloidal stability, loading capacity and encapsulation efficiency	Wu et al. [99]

## References

[1] Huh A.J., Kwon Y.J. (2011). “Nanoantibiotics”: A new paradigm for treating infectious diseases using nanomaterials in the antibiotics resistant era. J. Control. Release.

[2] Brooks B.D., Brooks A.E. (2014). Therapeutic strategies to combat antibiotic resistance. Adv. Drug Deliv. Rev..

[3] Pelgrift R.Y., Friedman A.J. (2013). Nanotechnology as a therapeutic tool to combat microbial resistance. Adv. Drug Deliv. Rev..

[4] Meade E., Slattery M.A., Garvey M. (2020). Bacteriocins, Potent Antimicrobial Peptides and the Fight against Multi Drug Resistant Species: Resistance Is Futile?. Antibiotics.

[5] Biswaro L.S., da Costa Sousa M.G., Rezende T.M.B., Dias S.C., Franco O.L. (2018). Antimicrobial Peptides and Nanotechnology, Recent Advances and Challenges. Front. Microbiol..

[6] Kumar P., Kizhakkedathu J.N., Straus S.K. (2018). Antimicrobial Peptides: Diversity, Mechanism of Action and Strategies to Improve the Activity and Biocompatibility In Vivo. Biomolecules.

[7] Martin-Serrano Á., Gómez R., Ortega P., de la Mata F.J. (2019). Nanosystems as Vehicles for the Delivery of Antimicrobial Peptides (AMPs). Pharmaceutics.

[8] Burrer R., Neuman B.W., Ting J.P., Stein D.A., Moulton H.M., Iversen P.L., Kuhn P., Buchmeier M.J. (2007). Antiviral effects of antisense morpholino oligomers in murine coronavirus infection models. J. Virol..

[9] Ikeda Y., Taira T., Sakai K., Sakai H., Shigeri Y., Imura T. (2018). Lipid Nanodisc Formation using Pxt-5 Peptide Isolated from Amphibian (Xenopus tropicalis) Skin, and its Altered Form, Modify-Pxt-5. J. Oleo Sci..

[10] Cavalieri F., Tortora M., Stringaro A., Colone M., Baldassarri L. (2014). Nanomedicines for antimicrobial interventions. J. Hosp. Infect..

[11] Diniz F.R., Maia R.C.A.P., Rannier L., Andrade L.N., Chaud M.V., da Silva C.F., Corrêa C.B., de Albuquerque Junior R.L.C., da Costa L.P., Souto E.B. (2020). Silver nanoparticles-composing alginate/gelatin hydrogel improves wound healing in vivo. Nanomaterials.

[12] Hissae Yassue-Cordeiro P., Zandonai C.H., Pereira Genesi B., Santos Lopes P., Sanchez-Lopez E., Garcia M.L., Camargo Fernandes-Machado N.R., Severino P., Souto E.B., da Silva C.F. (2019). Development of Chitosan/Silver Sulfadiazine/Zeolite Composite Films for Wound Dressing. Pharmaceutics.

[13] Zhu X., Radovic-Moreno A.F., Wu J., Langer R., Shi J. (2014). Nanomedicine in the Management of Microbial Infection—Overview and Perspectives. Nano Today.

[14] Souto E.B., Silva G.F., Dias-Ferreira J., Zielinska A., Ventura F., Durazzo A., Lucarini M., Novellino E., Santini A. (2020). Nanopharmaceutics: Part II—Production scales and clinically compliant production methods. Nanomaterials.

[15] Souto E.B., Silva G.F., Dias-Ferreira J., Zielinska A., Ventura F., Durazzo A., Lucarini M., Novellino E., Santini A. (2020). Nanopharmaceutics: Part I—Clinical Trials Legislation and Good Manufacturing Practices (GMP) of Nanotherapeutics in the EU. Pharmaceutics.

[16] Nordstrom R., Malmsten M. (2017). Delivery systems for antimicrobial peptides. Adv. Colloid Interface Sci..

[17] Bahar A.A., Ren D. (2013). Antimicrobial peptides. Pharmaceuticals.

[18] da Costa J.P., Cova M., Ferreira R., Vitorino R. (2015). Antimicrobial peptides: An alternative for innovative medicines?. Appl. Microbiol. Biotechnol..

[19] Narayana J.L., Chen J.Y. (2015). Antimicrobial Peptides: Possible Anti-Infective agents. Peptides.

[20] Mok W.W., Li Y. (2014). Therapeutic peptides: New arsenal against drug resistant pathogens. Curr. Pharm. Des..

[21] Singh S., Papareddy P., Morgelin M., Schmidtchen A., Malmsten M. (2014). Effects of PEGylation on membrane and lipopolysaccharide interactions of host defense peptides. Biomacromolecules.

[22] Mishra B., Basu A., Chua R.R.Y., Saravanan R., Tambyah P.A., Ho B., Chang M.W., Leong S.S.J. (2014). Site specific immobilization of a potent antimicrobial peptide onto silicone catheters: Evaluation against urinary tract infection pathogens. J. Mater. Chem. B.

[23] Water J.J., Smart S., Franzyk H., Foged C., Nielsen H.M. (2015). Nanoparticle-mediated delivery of the antimicrobial peptide plectasin against Staphylococcus aureus in infected epithelial cells. Eur. J. Pharm. Biopharm..

[24] Arias C.A., Murray B.E. (2012). The rise of the Enterococcus: Beyond vancomycin resistance. Nat. Rev. Microbiol..

[25] Spellberg B., Bartlett J.G., Gilbert D.N. (2013). The Future of Antibiotics and Resistance. New Engl. J. Med..

[26] Gullberg E., Cao S., Berg O.G., Ilback C., Sandegren L., Hughes D., Andersson D.I. (2011). Selection of Resistant Bacteria at Very Low Antibiotic Concentrations. PLoS Pathog..

[27] Cira N.J., Ho J.Y., Dueck M.E., Weibel D.B. (2012). A self-loading microfluidic device for determining the minimum inhibitory concentration of antibiotics. Lab Chip.

[28] D’Costa V.M., McGrann K.M., Hughes D.W., Wright G.D. (2006). Sampling the antibiotic resistome. Science.

[29] Davies J. (1996). Bacteria on the rampage. Nature.

[30] Spratt B.G. (1994). Resistance to antibiotics mediated by target alterations. Science.

[31] Chopra I., Roberts M. (2001). Tetracycline antibiotics: Mode of action, applications, molecular biology, and epidemiology of bacterial resistance. Microbiol. Mol. Biol. Rev..

[32] Diab R., Khameneh B., Joubert O., Duval R. (2015). Insights in Nanoparticle-Bacterium Interactions: New Frontiers to Bypass Bacterial Resistance to Antibiotics. Curr. Pharm. Des..

[33] Shimanovich U., Gedanken A. (2016). Nanotechnology solutions to restore antibiotic activity. J. Mater. Chem. B.

[34] Hajipour M.J., Fromm K.M., Ashkarran A.A., Jimenez de Aberasturi D., Ruiz de Larramendi I., Rojo T., Serpooshan V., Parak W.J., Mahmoudi M. (2012). Antibacterial properties of nanoparticles. Trends Biotechnol..

[35] Ganjian H., Nikokar I., Tieshayar A., Mostafaei A., Amirmozafari N., Kiani S. (2012). Effects of Salt Stress on the Antimicrobial Drug Resistance and Protein Profile of Staphylococcus aureus. Jundishapur J. Microbiol..

[36] Jayaraman R. (2009). Antibiotic resistance: An overview of mechanisms and a paradigm shift. Curr. Sci..

[37] Rai M., Ingle A.P., Gaikwad S., Gupta I., Gade A., da Silva S.S. (2016). Nanotechnology based anti-infectives to fight microbial intrusions. J. Appl. Microbiol..

[38] Sánchez-López E., Gomes D., Esteruelas G., Bonilla L., Lopez-Machado A.L., Galindo R., Cano A., Espina M., Ettcheto M., Camins A. (2020). Metal-Based Nanoparticles as Antimicrobial Agents: An Overview. Nanomaterials.

[39] Chen W.Y., Chang H.Y., Lu J.K., Huang Y.C., Harroun S.G., Tseng Y.T., Li Y.J., Huang C.C., Chang H.T. (2015). Self-Assembly of Antimicrobial Peptides on Gold Nanodots: Against Multidrug-Resistant Bacteria and Wound-Healing Application. Adv. Funct. Mater..

[40] Rai A., Pinto S., Velho T.R., Ferreira A.F., Moita C., Trivedi U., Evangelista M., Comune M., Rumbaugh K.P., Simoes P.N. (2016). One-step synthesis of high-density peptide-conjugated gold nanoparticles with antimicrobial efficacy in a systemic infection model. Biomaterials.

[41] Akrami M., Balalaie S., Hosseinkhani S., Alipour M., Salehi F., Bahador A., Haririan I. (2016). Tuning the anticancer activity of a novel pro-apoptotic peptide using gold nanoparticle platforms. Sci. Rep..

[42] Geilich B.M., van de Ven A.L., Singleton G.L., Sepúlveda L.J., Sridhar S., Webster T.J. (2015). Silver nanoparticle-embedded polymersome nanocarriers for the treatment of antibiotic-resistant infections. Nanoscale.

[43] Pazos E., Sleep E., Perez C.M.R., Lee S.S., Tantakitti F., Stupp S.I. (2016). Nucleation and Growth of Ordered Arrays of Silver Nanoparticles on Peptide Nanofibers: Hybrid Nanostructures with Antimicrobial Properties. J. Am. Chem. Soc..

[44] Salouti M., Mirzaei F., Shapouri R., Ahangari A. (2016). Synergistic Antibacterial Activity of Plant Peptide MBP-1 and Silver Nanoparticles Combination on Healing of Infected Wound Due to Staphylococcus aureus. Jundishapur J. Microbiol..

[45] Chaudhari A.A., Ashmore D., deb Nath S., Kate K., Dennis V., Singh S.R., Owen D.R., Palazzo C., Arnold R.D., Miller M.E. (2016). A novel covalent approach to bio-conjugate silver coated single walled carbon nanotubes with antimicrobial peptide. J. Nanobiotechnol..

[46] Braun K., Pochert A., Linden M., Davoudi M., Schmidtchen A., Nordstrom R., Malmsten M. (2016). Membrane interactions of mesoporous silica nanoparticles as carriers of antimicrobial peptides. J. Colloid Interface Sci..

[47] Syryamina V.N., Samoilova R.I., Tsvetkov Y.D., Ischenko A.V., De Zotti M., Gobbo M., Toniolo C., Formaggio F., Dzuba S.A. (2016). Peptides on the Surface: Spin-Label EPR and PELDOR Study of Adsorption of the Antimicrobial Peptides Trichogin GA IV and Ampullosporin A on the Silica Nanoparticles. Appl. Magn. Reson..

[48] Meira S.M.M., Jardim A.I., Brandelli A. (2015). Adsorption of nisin and pediocin on nanoclays. Food Chem..

[49] Barbosa G.P., Debone H.S., Severino P., Souto E.B., da Silva C.F. (2016). Design and characterization of chitosan/zeolite composite films—Effect of zeolite type and zeolite dose on the film properties. Mater. Sci. Eng. C.

[50] Hernandez-Delgadillo R., Velasco-Arias D., Diaz D., Arevalo-Niño K., Garza-Enriquez M., De la Garza-Ramos M.A., Cabral-Romero C. (2012). Zerovalent bismuth nanoparticles inhibit Streptococcus mutans growth and formation of biofilm. Int. J. Nanomed..

[51] Zhang W., Li Y., Niu J., Chen Y. (2013). Photogeneration of reactive oxygen species on uncoated silver, gold, nickel, and silicon nanoparticles and their antibacterial effects. Langmuir.

[52] Huynh K.A., Chen K.L. (2011). Aggregation kinetics of citrate and polyvinylpyrrolidone coated silver nanoparticles in monovalent and divalent electrolyte solutions. Environ. Sci. Technol..

[53] Rosi N.L., Giljohann D.A., Thaxton C.S., Lytton-Jean A.K., Han M.S., Mirkin C.A. (2006). Oligonucleotide-modified gold nanoparticles for intracellular gene regulation. Science.

[54] Cho E.C., Au L., Zhang Q., Xia Y. (2010). The effects of size, shape, and surface functional group of gold nanostructures on their adsorption and internalization by cells. Small.

[55] Rosemary M.J., MacLaren I., Pradeep T. (2006). Investigations of the antibacterial properties of ciprofloxacin@SiO2. Langmuir.

[56] Kitov P.I., Mulvey G.L., Griener T.P., Lipinski T., Solomon D., Paszkiewicz E., Jacobson J.M., Sadowska J.M., Suzuki M., Yamamura K. (2008). In vivo supramolecular templating enhances the activity of multivalent ligands: A potential therapeutic against the Escherichia coli O157 AB5 toxins. Proc. Natl. Acad. Sci. USA.

[57] Yavuz M.S., Cheng Y., Chen J., Cobley C.M., Zhang Q., Rycenga M., Xie J., Kim C., Song K.H., Schwartz A.G. (2009). Gold nanocages covered by smart polymers for controlled release with near-infrared light. Nat. Mater..

[58] Lin C.C., Yeh Y.C., Yang C.Y., Chen C.L., Chen G.F., Chen C.C., Wu Y.C. (2002). Selective binding of mannose-encapsulated gold nanoparticles to type 1 pili in Escherichia coli. J. Am. Chem. Soc..

[59] Nair S., Sasidharan A., Rani V.V.D., Menon D., Nair S., Manzoor K., Raina S. (2009). Role of size scale of ZnO nanoparticles and microparticles on toxicity toward bacteria and osteoblast cancer cells. J. Mater. Sci. Mater. Med..

[60] Kumar A., Vemula P.K., Ajayan P.M., John G. (2008). Silver-nanoparticle-embedded antimicrobial paints based on vegetable oil. Nat. Mater..

[61] Millenbaugh N.J., Baskin J.B., DeSilva M.N., Elliott W.R., Glickman R.D. (2015). Photothermal killing of Staphylococcus aureus using antibody-targeted gold nanoparticles. Int. J. Nanomed..

[62] de Azeredo H.M.C. (2013). Antimicrobial nanostructures in food packaging. Trends Food Sci. Technol..

[63] Xie S., Tao Y., Pan Y., Qu W., Cheng G., Huang L., Chen D., Wang X., Liu Z., Yuan Z. (2014). Biodegradable nanoparticles for intracellular delivery of antimicrobial agents. J. Control. Release.

[64] Soppimath K.S., Aminabhavi T.M., Kulkarni A.R., Rudzinski W.E. (2001). Biodegradable polymeric nanoparticles as drug delivery devices. J. Control. Release.

[65] Langer R., Folkman J. (1976). Polymers for the sustained release of proteins and other macromolecules. Nature.

[66] Murray J., Brown L., Langer R. (1984). Controlled release of microquantities of macromolecules. Cancer Drug Deliv..

[67] Zhang L., Pornpattananangkul D., Hu C.M.J., Huang C.M. (2010). Development of Nanoparticles for Antimicrobial Drug Delivery. Curr. Med. Chem..

[68] Santos-Magalhaes N.S., Furtado Mosqueira V.C. (2010). Nanotechnology applied to the treatment of malaria. Adv. Drug Deliv. Rev..

[69] Sosnik A., Carcaboso A.M., Glisoni R.J., Moretton M.A., Chiappetta D.A. (2010). New old challenges in tuberculosis: Potentially effective nanotechnologies in drug delivery. Adv. Drug Deliv. Rev..

[70] Vauthier C., Dubernet C., Fattal E., Pinto-Alphandary H., Couvreur P. (2003). Poly(alkylcyanoacrylates) as biodegradable materials for biomedical applications. Adv. Drug Deliv. Rev..

[71] Abeylath S.C., Turos E. (2008). Drug delivery approaches to overcome bacterial resistance to beta-lactam antibiotics. Expert Opin. Drug Deliv..

[72] Owens D.E., Peppas N.A. (2006). Opsonization, biodistribution, and pharmacokinetics of polymeric nanoparticles. Int. J. Pharm..

[73] Carbone C., Manno D., Serra A., Musumeci T., Pepe V., Tisserand C., Puglisi G. (2016). Innovative hybrid vs polymeric nanocapsules: The influence of the cationic lipid coating on the “4S”. Colloids Surf. B Biointerfaces.

[74] Carbone C., Musumeci T., Lauro M.R., Puglisi G. (2015). Eco-friendly aqueous core surface-modified nanocapsules. Colloids Surf. B Biointerfaces.

[75] Musumeci T., Bucolo C., Carbone C., Pignatello R., Drago F., Puglisi G. (2013). Polymeric nanoparticles augment the ocular hypotensive effect of melatonin in rabbits. Int. J. Pharm..

[76] Bonaccorso A., Musumeci T., Carbone C., Vicari L., Lauro M.R., Puglisi G. (2017). Revisiting the role of sucrose in PLGA-PEG nanocarrier for potential intranasal delivery. Pharm. Dev. Technol..

[77] Cheng J., Teply B.A., Sherifi I., Sung J., Luther G., Gu F.X., Levy-Nissenbaum E., Radovic-Moreno A.F., Langer R., Farokhzad O.C. (2007). Formulation of functionalized PLGA-PEG nanoparticles for in vivo targeted drug delivery. Biomaterials.

[78] Gu H.W., Ho P.L., Tong E., Wang L., Xu B. (2003). Presenting vancomycin on nanoparticles to enhance antimicrobial activities. Nano Lett..

[79] Rishi P., Bhogal A., Arora S., Pandey S.K., Verma I., Kaur I.P. (2015). Improved oral therapeutic potential of nanoencapsulated cryptdin formulation against Salmonella infection. Eur. J. Pharm. Sci..

[80] Forestier F., Gerrier P., Chaumard C., Quero A.M., Couvreur P., Labarre C. (1992). Effect of nanoparticle-bound Ampicillin on the survival of listeria-monocytogenes in mouse peritoneal-macrophages. J. Antimicrob. Chemother..

[81] Fontana G., Pitarresi G., Tomarchio V., Carlisi B., San Biagio P.L. (1998). Preparation, characterization and in vitro antimicrobial activity of ampicillin-loaded polyethylcyanoacrylate nanoparticles. Biomaterials.

[82] Fontana G., Licciardi M., Mansueto S., Schillaci D., Giammona G. (2001). Amoxicillin-loaded polyethylcyanoacrylate nanoparticles: Influence of PEG coating on the particle size, drug release rate and phagocytic uptake. Biomaterials.

[83] Sonia T.A., Sharma C.P. (2011). Chitosan and Its Derivatives for Drug Delivery Perspective. Adv. Polym. Sci..

[84] Teixeira M.D.C., Santini A., Souto E.B., Grumezescu A.M. (2017). Chapter 8-Delivery of Antimicrobials by Chitosan-Composed Therapeutic Nanostructures A2-Ficai, Anton. Nanostructures for Antimicrobial Therapy.

[85] Rivera M.C., Pinheiro A.C., Bourbon A.I., Cerqueira M.A., Vicente A.A. (2015). Hollow chitosan/alginate nanocapsules for bioactive compound delivery. Int. J. Biol. Macromol..

[86] Cerchiara T., Abruzzo A., di Cagno M., Bigucci F., Bauer-Brandl A., Parolin C., Vitali B., Gallucci M.C., Luppi B. (2015). Chitosan based micro- and nanoparticles for colon-targeted delivery of vancomycin prepared by alternative processing methods. Eur. J. Pharm. Biopharm..

[87] Piras A.M., Maisetta G., Sandreschi S., Esin S., Gazzarri M., Batoni G., Chiellini F. (2014). Preparation, physical-chemical and biological characterization of chitosan nanoparticles loaded with lysozyme. Int. J. Biol. Macromol..

[88] Sun Y., Liu Y., Liu W., Lu C.J., Wang L. (2015). Chitosan microparticles ionically cross-linked with poly(gamma-glutamic acid) as antimicrobial peptides and nitric oxide delivery systems. Biochem. Eng. J..

[89] Piras A.M., Maisetta G., Sandreschi S., Gazzarri M., Bartoli C., Grassi L., Esin S., Chiellini F., Batoni G. (2015). Chitosan nanoparticles loaded with the antimicrobial peptide temporin B exert a long-term antibacterial activity in vitro against clinical isolates of Staphylococcus epidermidis. Front. Microbiol..

[90] Coll Ferrer M.C., Dastgheyb S., Hickok N.J., Eckmann D.M., Composto R.J. (2014). Designing nanogel carriers for antibacterial applications. Acta Biomater..

[91] Pereira P., Pedrosa S.S., Correia A., Lima C.F., Olmedo M.P., Gonzalez-Fernandez A., Vilanova M., Gama F.M. (2015). Biocompatibility of a self-assembled glycol chitosan nanogel. Toxicol. In Vitro.

[92] Gomes A.P., Mano J.F., Queiroz J.A., Gouveia I.C. (2015). Incorporation of antimicrobial peptides on functionalized cotton gauzes for medical applications. Carbohydr. Polym..

[93] Imran M., Klouj A., Revol-Junelles A.M., Desobry S. (2014). Controlled release of nisin from HPMC, sodium caseinate, poly-lactic acid and chitosan for active packaging applications. J. Food Eng..

[94] d’Angelo I., Casciaro B., Miro A., Quaglia F., Mangoni M.L., Ungaro F. (2015). Overcoming barriers in Pseudomonas aeruginosa lung infections: Engineered nanoparticles for local delivery of a cationic antimicrobial peptide. Colloids Surf. B Biointerfaces.

[95] Jiang L.H., Xu D.W., Sellati T.J., Dong H. (2015). Self-assembly of cationic multidomain peptide hydrogels: Supramolecular nanostructure and rheological properties dictate antimicrobial activity. Nanoscale.

[96] Water J.J., Kim Y., Maltesen M.J., Franzyk H., Foged C., Nielsen H.M. (2015). Hyaluronic Acid-Based Nano gels Produced by Microfluidics-Facilitated Self-Assembly Improves the Safety Profile of the Cationic Host Defense Peptide Novicidin. Pharm. Res..

[97] Li X., Fan R., Tong A., Yang M., Deng J., Zhou L., Zhang X., Guo G. (2015). In situ gel-forming AP-57 peptide delivery system for cutaneous wound healing. Int. J. Pharm..

[98] Krivorotova T., Cirkovas A., Maciulyte S., Staneviciene R., Budriene S., Serviene E., Sereikaite J. (2016). Nisin-loaded pectin nanoparticles for food preservation. Food Hydrocoll..

[99] Wu C.H., Wu T.T., Fang Z.X., Zheng J.W., Xu S., Chen S.G., Hu Y.Q., Ye X.Q. (2016). Formation, characterization and release kinetics of chitosan/gamma-PGA encapsulated nisin nanoparticles. RSC Adv..

[100] Ghobril C., Grinstaff M.W. (2015). The chemistry and engineering of polymeric hydrogel adhesives for wound closure: A tutorial. Chem. Soc. Rev..

[101] Cabral J.D., Kalia S. (2016). Antimicrobial Polymeric Hydrogels. Polymeric Hydrogels as Smart Biomaterials.

[102] Souto E.B., Doktorovova S. (2009). Chapter 6-Solid lipid nanoparticle formulations pharmacokinetic and biopharmaceutical aspects in drug delivery. Methods Enzymol..

[103] Doktorovova S., Kovacevic A.B., Garcia M.L., Souto E.B. (2016). Preclinical safety of solid lipid nanoparticles and nanostructured lipid carriers: Current evidence from in vitro and in vivo evaluation. Eur. J. Pharm. Biopharm..

[104] Doktorovova S., Silva A.M., Gaivao I., Souto E.B., Teixeira J.P., Martins-Lopes P. (2014). Comet assay reveals no genotoxicity risk of cationic solid lipid nanoparticles. J. Appl. Toxicol..

[105] Doktorovova S., Souto E.B., Silva A.M. (2014). Nanotoxicology applied to solid lipid nanoparticles and nanostructured lipid carriers-a systematic review of in vitro data. Eur. J. Pharm. Biopharm..

[106] Souto E.B., Almeida A.J., Müller R.H. (2007). Lipid Nanoparticles (SLN^®^, NLC^®^) for Cutaneous Drug Delivery:Structure, Protection and Skin Effects. J. Biomed. Nanotechnol..

[107] Almeida A.J., Souto E. (2007). Solid lipid nanoparticles as a drug delivery system for peptides and proteins. Adv. Drug Deliv. Rev..

[108] Muller R.H., Runge S., Ravelli V., Mehnert W., Thunemann A.F., Souto E.B. (2006). Oral bioavailability of cyclosporine: Solid lipid nanoparticles (SLN) versus drug nanocrystals. Int. J. Pharm..

[109] Muller R.H., Runge S.A., Ravelli V., Thunemann A.F., Mehnert W., Souto E.B. (2008). Cyclosporine-loaded solid lipid nanoparticles (SLN): Drug-lipid physicochemical interactions and characterization of drug incorporation. Eur. J. Pharm. Biopharm..

[110] Souto E.B., Souto S.B., Zielinska A., Durazzo A., Lucarini M., Santini A., Horbańczuk O.K., Atanasov A.G., Marques C., Andrade L.N. (2020). Perillaldehyde 1,2-epoxide loaded SLN-tailored mAb: Production, physicochemical characterization and in vitro cytotoxicity profile in MCF-7 cell lines. Pharmaceutics.

[111] Souto E.B., Doktorovova S., Campos J.R., Martins-Lopes P., Silva A.M. (2019). Surface-tailored anti-HER2/neu-solid lipid nanoparticles for site-specific targeting MCF-7 and BT-474 breast cancer cells. Eur. J. Pharm. Sci..

[112] Fumakia M., Ho E.A. (2016). Nanoparticles Encapsulated with LL37 and Serpin A1 Promotes Wound Healing and Synergistically Enhances Antibacterial Activity. Mol. Pharm..

[113] Garcia-Orue I., Gainza G., Girbau C., Alonso R., Aguirre J.J., Pedraz J.L., Igartua M., Hernandez R.M. (2016). LL37 loaded nanostructured lipid carriers (NLC): A new strategy for the topical treatment of chronic wounds. Eur. J. Pharm. Biopharm..

[114] Lewies A., Wentzel J.F., Jordaan A., Bezuidenhout C., Du Plessis L.H. (2017). Interactions of the antimicrobial peptide nisin Z with conventional antibiotics and the use of nanostructured lipid carriers to enhance antimicrobial activity. Int. J. Pharm..

[115] Gide M., Nimmagadda A., Su M., Wang M., Teng P., Li C., Gao R., Xu H., Li Q., Cai J. (2018). Nano-Sized Lipidated Dendrimers as Potent and Broad-Spectrum Antibacterial Agents. Macromol. Rapid Commun..

[116] Dong X., Liang W., Meziani M.J., Sun Y.-P., Yang L. (2020). Carbon Dots as Potent Antimicrobial Agents. Theranostics.

[117] Zhong D., Zhuo Y., Feng Y., Yang X. (2015). Employing carbon dots modified with vancomycin for assaying Gram-positive bacteria like Staphylococcus aureus. Biosens. Bioelectron..

[118] Pramanik A., Jones S., Pedraza F., Vangara A., Sweet C., Williams M.S., Ruppa-Kasani V., Risher S.E., Sardar D., Ray P.C. (2017). Fluorescent, Magnetic Multifunctional Carbon Dots for Selective Separation, Identification, and Eradication of Drug-Resistant Superbugs. ACS Omega.

